# Holocord oligodendroglioma with intracranial extension in a young adult: a case report and review of literature

**DOI:** 10.2217/cns-2017-0012

**Published:** 2018-02-02

**Authors:** Romulus Emmanuel H Cruz, Ranhel C De Roxas, Carmela Concepcion A Sales-Callangan, Roland Dominic G Jamora

**Affiliations:** 1 Department of Neurosciences, College of Medicine – Philippine General Hospital, University of the Philippines Manila, Taft Avenue, Ermita, Manila, 1000, Philippines

**Keywords:** holocord tumors, intramedullary spinal cord tumors, spinal gliomas, spinal oligodendroglioma, widespread spinal tumors

## Abstract

Widespread primary spinal oligodendrogliomas are a rare variety of tumors that usually affect children. Currently, there are only two adult cases reported worldwide. We report the first case of primary holocord oligodendroglioma with intracranial extension in a young adult female. The patient presented with a 4-month history of fluctuating hemiparesis of the left upper extremity eventually becoming quadriplegic after 1 month. Imaging findings revealed a contrast-enhancing holocord neoplasm spanning from the cervical region to the conus medullaris and with extension to the lower medulla. The patient succumbed to severe pneumonia after 1 month of admission. An autopsy was done and the histopathologic findings were consistent with oligodendroglioma.

**Practice points**

- Widespread primary spinal oligodendogliomas in adults are rare neoplasms with only two documented adult cases worldwide.
- These neoplasms usually affect the pediatric population and its occurrence among adults usually results in poor outcomes.
- It may present with a history of fluctuating hemiparesis which will eventually evolve to quadriplegia.
- Imaging findings may reveal a contrast-enhancing holocord neoplasm spanning from the cervical region to the conus medullaris and with extension to the lower medulla.

Primary holocord tumors are rare variety of spinal cord neoplasms which are intramedullary neoplasms usually distributed over the cervicomedullary junction to the conus medullaris or spanning 19–20 contiguous cord segments [1,2]. The most common holocord tumors identified are astrocytoma, ependymomas and glioblastoma [1,3,4].

Oligodendroglioma of the spinal cord is an uncommon variety of tumor consisting only 2% of all spinal cord tumors [5]. To date, only 53 cases of primary spinal oligodendroglioma were documented worldwide [6]. Currently, there are only eight cases of widespread primary spinal oligodendroglioma, both in the pediatric and adult age group, with only two cases meeting the ‘holocord’ definition and none of these have intracranial extension [2,6–10]. We report the first case of primary holocord spinal tumor in a young adult female with extension to the medullary region.

## Case

We report a case of a 23-year-old female who presented with a 4-month history of fluctuating weakness of the right upper and lower extremities associated with a band-like sensation of the trunk along with bowel and bladder disturbances. There was no consult done. One month prior to her admission, she noted electric-like sensation on her nape, which was worsened by neck movement. The weakness eventually progressed to the left lower extremity causing difficulty in ambulation. The patient eventually became quadriparetic and needed to be assisted in all activities of daily living. She also developed dysphonia, dysphagia, difficulty of breathing and projectile vomiting that prompted consult to our institution.

The patient was hemodynamically stable upon admission. There were no spinal deformities noted. The sphincter tone was lax. Higher cortical function was normal. On cranial nerve examination, there was weak gag and shoulder shrug bilaterally. Manual motor testing revealed 2–3/5 on both upper extremities and left lower extremity and strength of 4/5 on the right lower extremity. Hypesthesia was also described at the C4 dermatomal level and dissociated sensory loss was also noted. The patient was hyper-reflexic on both the upper and lower extremities along with bilateral extensor toe sign. The patient's neck was paratonic.

**Figure 1.**
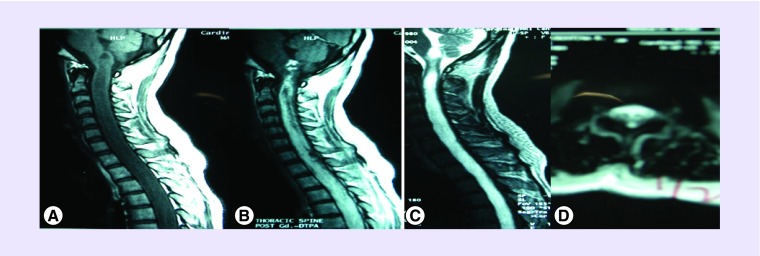
**MRI findings of the patient.** **(A)** MRI midsagittal T1-weighted image of the cervicomedullary junction and thoracic spine showing a hypointense long segment with expansile mass with a maximal diameter of 1.7 cm measured at the lower cervical region. **(B)** MRI with intense enhancement after administration of gadolinium. **(C)** T2-weighted image showing the intramedullary mass with slight heterogenous signal intensity from the level of the medulla down to the **(D)** axial cut section of the conus medullaris.

Initial work-up included a cranio-spinal magnetic resonance imaging (MRI) revealing a long expansile contrast-enhancing mass involving the entire spinal cord, from the level of the cervical region to the conus medullaris with extension to the lower medulla oblongata (Figure 1A–D). The patient was started on dexamethasone, which provided minimal relief of symptoms. She was also referred to the neurosurgery service. The patient was advised surgery, however, on the 20th hospital day, the patient's symptoms progressed to quadriplegia and respiratory failure. She subsequently developed healthcare-associated pneumonia succumbing to septicemia.

**Figure 2.**
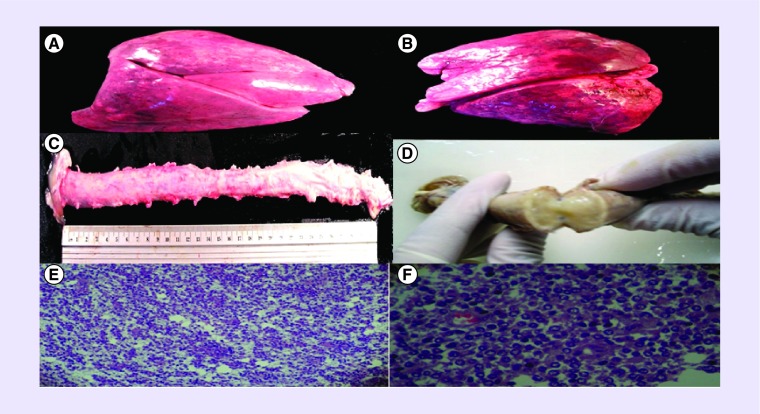
**Autopsy findings of the patient.** **(A)** The right lung weighs 750 g and **(B)** the left lung weighs 450 g *in situ*, both lungs are red to maroon in color, all lobes are consolidated. **(C)** The spinal cord is 32 cm in length with cream-white smooth outer surface. **(D)** Cut section showing that the entire spinal cord was swollen and replaced by the mucoid, gelatinous material. **(E)** Photomicrograph of the tumor showing small, round cells, with slightly ovoid nuclei, occasional clumping of chromatin, cytoplasmic fibrillary process, perinuclear cytoptoplasmic clearing and vacuolation, small capillaries coursing through the tumor at low power field (10× magnification). **(F)** Photomicrograph at high power field (40× magnification).

On autopsy, all lobes of the lungs were consolidated secondary to pneumonia (Figure 2A & B). There was swelling of the entire spinal cord with white to yellow mucoid material coming out from the central canal (Figure 2C). Cut sections showed that the entire spinal cord and caudal medullary region were replaced by mucoid material (Figure 2D). The rest of the examination was unremarkable. Histopathologic examination of the spinal cord revealed a cellular tumor arranged around the central canal of the cord. The cells are generally small, with round to slightly ovoid nuclei. The chromatin materials are generally vesicular with occasional clumping seen. Fibrillary processes were noted in the cytoplasm (Figure 2E & F). Immunohistochemical studies were done. S100 showed staining of the cytoplasmic globules with weak staining of intact cytoplasms. GFAP, synaptophysin, κ, λ and leucocyte common antigen had negative results. Further genetic characterization of the tumor such as *IDH, 1p/19q codeletion, MGMT, EGFR and TP53* mutations was not done because of its unavailability in the institution but its histologic and immunochemical features are consistent with holocord oligodendroglioma.

## Discussion

Widespread primary spinal cord oligodendrogliomas are rare spinal cord tumors with only two documented cases in adults worldwide from 1968 to 2016 [6,10]. Table 1 summarizes the features of the reported cases. We document the first case of the holocord type with intracranial extension. Verbal and written consent was obtained by the authors for this case report.

**Table 1.** **Clinical features of patients with widespread spinal oligodendroglioma.**

**Study**	**Year**	**Age**	**Location**	**Clinical findings**	**Neuroimaging**	**Surgery**	**Adjuvant therapy**	**Outcome**	**Ref.**
Tobias *et al.*	2008	24M	C1-T12	Not described	Not described	Staged laminoplasty	RT	Improved	[10]

Tunthanathip *et al.*	2016	46M	C2-T4	Progressive neck pain, hemiparesis	T1 isointense, T2 hyperintense, gadolinium-enhancing heterogenous intramedullary mass 12.5 cm in length	Laminectomy	RT	Died after 3 years	[6]

Present case	2017	23F	Cervicomedullary region to the conus	Fluctuating hemiparesis, hyperreflexia and sensory deficits	T1 hypointense, T2 hyperintense with slight heterogenous signal with intense enhancement after administration of long gadolinium, long segment expansile mass	None	None	Died after 5 months	–

F: Female; M: Male; RT: Radiotherapy.

Extensive primary spinal oligodendrogliomas usually affect the pediatric male population with ages ranging from 38 months to 16 years and male-to-female ratio of 6:1 [2,6–10]. There are only two cases occurring in young adults including ours [10]. The index case is also the first adult female documented.

Spinal cord oligodendrogliomas usually occur in a few spinal segments mostly affecting the cervical region and a holocord distribution usually affects only 3% of the cases [8]. ‘Holocord’ tumors are intramedullary tumors usually distributed over the cervicomedullary junction to the conus medullaris or spanning 19–20 contiguous cord segments [1,2]. Among the three cases reviewed in this series, only two met the strict criteria for holocord distribution, including the index case. The other case, which did not meet the ‘holocord’ criteria despite its widespread extent, still showed the usual predilection over the cervicothoracic region. The clinical manifestations are dependent on the location of the tumor and the usual clinical findings include sensory deficits and bilateral extremity weakness, which can be fluctuating in occurrence as manifested by our patient.

MRI is the imaging of choice in the diagnosis of intraspinal tumors. The common findings associated with holocord and extensive spinal oligodendroglioma include hypo- to iso-intensity on T1 and high-signal intensity on T2. Enhancement may be heterogenous or irregular with gadolinium infusion [2,8,9]. These are consistent with the findings observed in the index case. Heterogenous enhancement is attributed to the high amount of calcium and may also be explained at a molecular level by the *1p/19q* codeletion which are both pathognomonic features of this neoplasm [12]. Cystic degeneration is common in this form of spinal neoplasm and may be a marker of a high grade tumor [2,13]. Calcifications may also be visualized in 28–40% of the cases [14]. Other ancillary diagnostics which may be used include spinal radiography and computed tomography myelography. It has been shown that vertebral scalloping, erosion of the peduncles and widening of the spinal vertebral canal are features observed in patients with extensive spinal oligodendrogliomas, the latter observed in the case reported [7,9,11]. These changes maybe secondary to plasticity that is readily observable among young patients.

In the past, when spinal MRI was not yet available, lumbar puncture was of diagnostic value. It has been shown that cerebrospinal fluid protein was elevated and cytology was positive in more than 50% of the cases [11]. In the index case, lumbar puncture was not performed because an MRI was readily available for diagnosis. Definitive diagnosis is still through biopsy. Just as the other cases discussed in this series, swelling of the spinal cord was observed. Gross pathologic examination of the spinal cord of the index case revealed yellow, mucoid material coming out of the central canal. Other gross pathologic features reported were, friable, hemorrhagic and infiltrative tumor with gelatinous consistency [5,8,9]. Tumoral bleed and peritumoral cysts were also observed in some cases [2,11].

In general, spinal oligodendrogliomas are WHO grade II tumors [15]. Consistent with other cases of extensive spinal oligodendroglioma, histopathologic features of the index case revealed a high tumor cellularity, with round to oval nuclei, hyperchromatic nuclei and with occasional clumping of the chromatin material as shown in Figure 1 [5,9]. The anaplastic type has also been observed in a number of cases and usually confers a poorer prognosis [9,10,16,17].

Metastasis of spinal oligodendroglioma is not uncommon and includes sites such as, the spinal leptomeninges, ventricles, hypophysis and the pons [11,13,17]. In one study describing cases of spinal oligodendroglioma, several cases were shown to have distant metastasis to the cerebral parenchyma, ventricles and ependyma [11]. Several studies also discussed the occurrence of cerebral and ventricular spread of primary spinal anaplastic oligodendroglioma [13,17]. There are no cases prior to this report that document direct contiguity of the holocord tumor to the intracranial structures. A possible mechanism of spread may be via the cerebrospinal fluid system, and in some cases, through surgical manipulation [17]. Also, the local aggressiveness of this tumor may explain its diffuse infiltration of the entire cord and the medulla as observed in the index case.

Prior to the advent of modern neuroimaging and electrophysiologic techniques, the management of tumors with extensive primary spinal cord tumors has been a challenge to every neurosurgeon. The introduction of sophisticated neurosurgical techniques and instruments has resulted in the improvement in the outcomes of patients. Radical dissection is still the procedure of choice for spinal oligodendrogliomas; however, for holocord tumors, this may be difficult. In the two cases of extensive primary spinal oligodendrogliomas reported, one underwent laminectomy while the other one underwent two-staged laminoplasty. It appears that staged laminoplasty confers an advantage to laminectomy because the reported case who underwent this surgery improved with some residual symptoms while the one who underwent laminectomy died, although the patient who died had a histopathologic diagnosis of anaplastic oligodendroglioma [10]. Radiotherapy, although controversial, may result in improved outcomes among patients with holocord tumors. Both patients described in this series underwent radiotherapy although only one had an improved outcome and with no tumor recurrence after [15]. For our patient, surgical intervention was advised but was not done because of marked septicemia during the early course of admission. Chemotherapy has not been elucidated for spinal oligodendroglioma. However, case reports have described chemotherapeutic agents such as carboplatin and temozolamide may improve the survival of patients with disseminated and recurrent disease [13].

The prognosis of patients with extensive spinal oligodendroglioma, especially that of the holocord type, is dismal if without treatment. The life expectancy is usually 5–9 months from the onset of symptoms [7]. Other poor prognostic factors observed in this series include histologic findings of anaplastic type, diffuse spread and older age of onset of symptoms. In our patient, the lack of surgical intervention precluded the poor outcome.

## Conclusion

Widespread primary spinal oligodendogliomas in adults are rare neoplasms with only two documented cases worldwide. We report the first of the holocord type with intracranial extension. These neoplasms usually affect the pediatric population and its occurrence among adults usually results in poor outcomes.
